# Sjogren's Syndrome: Clinical Benefits of Low-dose Naltrexone Therapy

**DOI:** 10.7759/cureus.4225

**Published:** 2019-03-11

**Authors:** Scott Zashin

**Affiliations:** 1 Rheumatology, University of Texas Southwest Medical Center, Dallas, USA

**Keywords:** sjogren syndrome, low dose naltrexone, joint pain, fatigue, autoimmune diseases

## Abstract

Sjogren’s Syndrome is a chronic autoimmune disorder that causes the inflammation of the lacrimal and salivary glands, resulting in dryness of the eyes and mouth. In addition, fatigue and musculoskeletal pain, often described as aching, is very common. Treatment directed toward alleviating the fatigue and pain associated with Sjogren’s is currently very limited. This report describes a case of a 47-year-old female with suspected Sjogren's based on long-standing dry eyes, dry mouth, joint pain, fatigue, elevated measures of inflammation, and a positive rheumatoid factor. She failed standard therapy but improved clinically with low-dose naltrexone therapy.

## Introduction

Low-dose naltrexone (LDN) is a unique compound that has pain-relieving and anti-inflammatory properties. Limited studies have shown benefit in helping relieve the pain in patients with fibromyalgia and improving disease activity in autoimmune conditions such as inflammatory bowel disease and multiple sclerosis. As a result, it seems reasonable that the medication might be useful in Sjogren's Syndrome, an autoimmune condition that is associated with pain and inflammation.

## Case presentation

A 47-year-old female was diagnosed with Sjogren’s Syndrome six years ago by another rheumatologist, based on her history of eye and mouth dryness. She was found to have a negative rheumatoid factor at that time, but her sedimentation rate by modified Westergren (erythrocyte sedimentation rate, ESR) was recorded as low as 48 and as high as 61 (normal: less than 20 mm/h). Her C-reactive protein (CRP) was 1.74 (normal less than 0.80 mg/dl). Two years ago, she saw a second rheumatologist who agreed with the diagnosis of Sjogren’s Syndrome. At that time, her rheumatoid factor was now elevated at 69 IU/ml (normal: less than 14 IU/m/). Her antinuclear antibody (ANA) and Sjogren antibodies (SS-A and SS-B) were absent. Her anti-CCP antibody and 14.3.3 ETA protein were normal. Her ESR was 48 and her CRP was 1.42. Past medical history included a diagnosis of fibromyalgia. She also had a history of breast cancer that had been in remission for 20 years, a generalized seizure disorder, and elevated liver tests with normal biopsy. Additional medical issues included symptoms of neuropathy, anxiety, and depression. A prior sleep study did not reveal evidence of sleep apnea. She first came to see this author 18 months ago, seeking another opinion, with complaints of fatigue, severe musculoskeletal pain, as well as dryness of her eyes and mouth.

Her daily medications to help with her symptoms of Sjogren’s Syndrome and fibromyalgia included Lexapro, Restasis, meloxicam 15 mg, vitamin D3, magnesium, tramadol 100 mg daily prn, salagen 5 mg tid prn, and hydroxychloroquine 400 mg daily. Her exam demonstrated widespread trigger points affecting both sides of her body, above and below her waist. Her blood work was remarkable for an ESR of 37 and a CRP of 0.77. Her alanine aminotransferase (ALT) was 40 U/L (normal 6-29 U/L).

She elected to try low-dose naltrexone (LDN), which was compounded using a short-acting filler and started at 1.5 mg daily with instructions to increase the medication weekly by 1.5 mg. She came back to see me two weeks after starting the medication and was taking 3 mg daily. She stated that she felt terrific. Her lab was remarkable for a normal ESR of 25 and a CRP of 2.33. Her ALT was normal.

She was seen in follow-up 16 months ago and remained on 3 mg of naltrexone. She felt well but complained of neuropathic pain. Her ESR was now 20. CRP was not ordered. Her ALT was 31 U/L. She was seen in follow-up 14 months ago and remained on 3 mg of naltrexone and continued to feel well without stiffness or pain. She noted that, previously, it would take her all day to feel better. Her ESR remained at 20 and CRP was only minimally increased at 0.87.

She was then seen in follow-up 11 months ago with complaints of increased achiness. She had widespread tender points. She was given a short course of corticosteroids with symptomatic improvement in place of meloxicam. Naltrexone was increased on that visit to 4.5 mg. On the day of that visit, her ESR was 40 and CRP was 25.7 ( current normal value less than 8 mg/L). She was again seen in follow-up nine months ago, doing well on 4.5 mg of naltrexone. Hydroxychloroquine was discontinued a few weeks earlier due to a prolonged QTc interval. Her ESR was back down to 20 and CRP was down to 10.9.

Overall, the patient noted significant clinical benefit with her fatigue and pain within two weeks of starting low-dose naltrexone but no significant change in her dry eyes or mouth. She continues to do well on low-dose naltrexone four months after stopping hydroxychloroquine due to the electrocardiogram (EKG) abnormalities. While her symptoms improved, what is most interesting about this case is that her clinical improvement was associated with an improvement in her inflammatory markers.

## Discussion

In the initial pilot study that used low-dose naltrexone in the treatment of fibromyalgia, the baseline sedimentation rate was a significant predictor of clinical response to LDN [1]. It was of interest to note that this patient’s dramatic clinical response to LDN correlated with an improvement in her ESR. It is postulated that low-dose naltrexone has a beneficial effect on the immune system due to the following mechanisms.

Low-dose naltrexone blocks mu-, delta-, and other opioid receptors. These receptors are present in the cells of the immune system. This inhibition may result in an upregulation of endorphins, which in addition to decreasing pain, may have a beneficial effect on the immune abnormalities by suppressing cell growth [2].

Low-dose naltrexone inhibits microglia activity. Microglia are immune cells in the central nervous system that, when stimulated, produce inflammatory products that may be associated with pain, fatigue, cognitive dysfunction (brain fog) sleep, and mood disorders. Low-dose naltrexone inhibits toll-like receptors that are found in microglia cells. As a result, the production of inflammatory substances declines with resulting symptomatic improvement [3-4]. The inhibition of these toll-like receptors has been postulated to be responsible for the effectiveness of hydroxychloroquine, a standard therapy in diseases such as Sjogren’s Syndrome and systemic lupus.

Overall, LDN is well-tolerated. The 50 mg standard dose of naltrexone is Food and Drug Administration (FDA) approved in the treatment of alcohol dependence and for inhibiting the effects of opioids. Low-dose naltrexone is sometimes used to help alleviate the symptoms of patients with chronic conditions, including fibromyalgia [1], Crohn’s disease [5-6], and multiple sclerosis [7]. The use of LDN at this low dose and for these indications is considered “off-label” use. In other words, it has not undergone the rigorous testing needed to get the approval of the FDA. Side effects include but are not limited to vivid dreams (most patients take the medication in the evening, but morning dosing of the medication may help with this issue). Patients may experience a reduction in pain relief from narcotics for at least six to 24 hours after taking low-dose naltrexone. In addition, low-dose naltrexone should not be used in patients who are currently receiving opioid analgesics due to the possibility of acute opioid withdrawal. The medication should be avoided until it is felt that the narcotics are out of the patient’s system. Those patients taking thyroid replacement may require a lower amount of thyroid medication so periodic monitoring is indicated. The elevation of liver enzymes is a potential risk with naltrexone treatment but is not felt to be common with low-dose therapy. Other potential side effects may include but are not limited to gastrointestinal disturbances, such as stomach cramps and diarrhea, agitation, anxiety, flu-like symptoms, and headaches. The drug should be compounded with short-acting fillers, so calcium carbonate should be avoided. The most common starting dose is 0.5 mg daily in the evening and increased weekly up to a target dose of 4.5 mg. The drug can be started at higher doses. The drug should be stopped at a minimum of 24 hours prior to the time narcotics may be needed for pain relief for a scheduled surgical procedure. In my practice, I will ask patients to temporarily discontinue low-dose naltrexone 72 hours in advance of taking narcotics if the patient is new to therapy, to ensure pain relief [8-11].

## Conclusions

Based on a Medline review, this is the first peer-reviewed case report of a patient with Sjogren's Syndrome who was treated with low-dose naltrexone and obtained clinical benefits. As a result of this case, further study is needed to determine if low-dose naltrexone will subsequently prove to be a useful medication for treating Sjogren’s Syndrome.
